# EFFECT OF HEARING LOSS TREATMENT ON ACCELEROMETRY-MEASURED DAILY PHYSICAL ACTIVITY

**DOI:** 10.1093/geroni/igae098.0610

**Published:** 2024-12-31

**Authors:** Jennifer Schrack, Amal Wanigatunga

**Affiliations:** Johns Hopkins Bloomberg School of Public Health, Baltimore, Maryland, United States; Johns Hopkins Bloomberg School of Public Health, Baltimore, Maryland, United States

## Abstract

Hearing impairment is widespread in older adults and associated with lower physical activity, but whether hearing loss treatment reduces or attenuates age-related decline in physical activity as individuals become more aware of -and interactive with- their surroudings is unknown. This study tested the effect of a hearing intervention vs. successful aging health education control on quantities and patterns of wrist-worn, accelerometer-derived daily physical activity over 3-years among older adults with hearing loss, a pre-specified exploratory outcome in the Aging and Cognitive Health Evaluation in Elders Study. Two-level linear mixed effects models with an interaction term (intervention x time) were used to test if 3-year change in physical activity outcomes differed by intervention assignment. Function-on-scalar regression (FOSR) models were used to examine potential intervention time-of-day differences in physical activity. Participants were (mean±SD) aged 76±6 years, 89% White, 52% female, and 53% had a Bachelor’s degree or higher. At baseline, mean activity counts/day were 1.86x106±810,889, active minutes/day were 382.3±160.5, and activity fragmentation was 23.8%±8.2%. At year 3 of follow-up, activity counts/day and active minutes/day declined (β [95%CI]) (-49,584 [-61,873, -37,294] and -8.7 [-11.4, -6.1]), respectively, and activity fragmentation increased (0.18% [0.01, 0.35%]). Results did not differ by intervention assignment. FOSR models showed no difference in time-of-day activity by intervention assignment. Results suggest the hearing intervention did not attenuate 3-year declines in physical activity in older adults with hearing loss. Findings may have been attenuated by the content of the successful aging program (single-session on physical activity), or relatively short follow-up time.

